# Relation Between Reading Performance and White-Matter Alteration and Reorganization in Neurosurgical Patients

**DOI:** 10.3389/fneur.2020.563259

**Published:** 2020-12-14

**Authors:** Elisa Cargnelutti, Marta Maieron, Tamara Ius, Miran Skrap, Barbara Tomasino

**Affiliations:** ^1^Scientific Institute, IRCCS E. Medea, Dipartimento/Unità Operativa Pasian di Prato, Udine, Italy; ^2^Struttura Organizzativa Complessa (SOC) Fisica Sanitaria, Azienda Sanitaria Universitaria Friuli Centrale (ASU FC), Udine, Italy; ^3^Struttura Organizzativa Complessa (SOC) Neurochirurgia, Azienda Sanitaria Universitaria Friuli Centrale (ASU FC), Udine, Italy

**Keywords:** reading, diffusion tensor imaging (DTI), neurosurgical patients, functional recovery, plasticity, direct arcuate fasciculus

## Abstract

Reading abilities and diffusion tensor imaging (DTI) parameters were retrospectively analyzed in a group of neurosurgical patients to investigate (Study 1) the role of white matter—in particular the arcuate fasciculus (AF)—in preserved vs. impaired reading; 4 months after surgery, we explored the plasticity processes (Study 2). Study 1 involved 40 patients with brain glioma (23 low-grade and 17 high-grade gliomas). We compared preoperative DTI parameters of language-related fascicles between patients who developed a reading impairment after surgery (*n* = 23) and patients with preserved reading (*n* = 17). Besides lower fractional anisotropy (FA), patients with impaired reading also displayed lower number and density of streamlines of a direct (i.e., directly connecting temporal and frontal lobes) AF segment. In Study 2, we longitudinally tested at follow-up-when reading performance had generally improved-13 patients diagnosed with low-grade glioma. The most relevant finding was a significant increase in length of streamlines of the direct AF segments in both hemispheres. From a neurosurgical perspective, our preliminary findings suggest the clinical importance of sparing direct AF segments for the involvement they showed in reading; however, the results also suggest the reorganization potential of these segments, possibly compensating of the right homologs as well.

## Introduction

Reading is a compound multistep process which relies on two main routes [e.g., (1, 2)]: the lexical-semantic ventral route, mediating direct access from word shape to meaning, and the sublexical dorsal route, driving grapheme-to-phoneme mapping. In patients with glioma, reading deficits are possible consequences of tumor growth, but they may also be due to surgery [e.g., (3, 4)]: In these patients, direct electrostimulation performed during neurosurgery showed that, despite a high reading localization variability, the sites whose stimulation induced reading impairments were located principally in the inferior parietal cortex and along the temporal lobe [e.g., (5, 6)].

Reading deficits were also related with altered subcortical white-matter pathways, although evidence mainly comes from developmental dyslexia. Many studies reported the crucial role of the inferior longitudinal fasciculus (ILF)—in particular for orthographic information processing [e.g., (7)]—the inferior fronto-occipital fasciculus (IFOF), involved in both orthographic and semantic processing [for developmental dyslexia, see (8)]—and the perisylvian bundles pertaining to arcuate fasciculus [AF; for developmental dyslexia, see (9–12)]. Also, the uncinate fasciculus (UF) appeared to be somewhat involved in reading, for instance for its role in semantics [e.g., (13–15); but see (16)].

Regarding the role of white-matter fascicles in postoperative reading attainment, Zemmoura et al. (4) reported deficits in both irregular word and pseudoword reading following resection of the terminations in the inferior temporal cortex of the left posterior AF. The authors also reported a global reading impairment caused by a resection of the posterior (but not anterior) ILF, in particular of fibers connecting the visual cortex to the Visual Word Form Area (in occipito-temporal cortex).

In the present study, we addressed the link between white-matter tracts and reading abilities in a population of patients undergoing surgery for a glioma and further explored a potential white-matter plasticity. The final aim was to provide a report of the fascicles that proved to be essential for reading and fascicles that could instead be compensated, in order to guide safe neurosurgery. We were particularly interested in exploring the role of AF and related segments. Thus, we first analyzed (Study 1) pre-surgery DTI data in a group of 40 patients and explored whether there were differences between patients who developed a postoperative reading impairment and patients showing optimal performance. We then investigated potential neuroplasticity processes after surgery (Study 2) by longitudinally monitoring a subgroup of patients with slowly growing lesions. We also aimed to inspect the role and potential changes of homolog white-matter structures in the healthy hemisphere.

The majority of studies investigating brain plasticity in gliomas addressed preoperative plasticity processes (i.e., associated with tumor growth) and mainly focused on gray matter and associated functional activation. Some of these studies reported a compensatory involvement of the contralesional hemisphere in response to glioma growth [e.g., (17, 18)]. Postoperative plasticity is poorly explored, in particular with respect to white matter. Further, it is still contended whether plasticity may occur at the white-matter level, although a potential subcortical plasticity has been reported [see (19)]. Nevertheless, preserving white-matter integrity is crucial for shaping cortical plasticity [e.g., (20)]. For instance, it has been shown that disruption of the anterior part of the ILF did not produce permanent language deficits because fascicles such as the IFOF could take over that function; [(21); see also (22, 23)]. In light of these findings, we explored whether significant changes took place between pre and postoperative DTI assessments in the investigated fascicles and whether these changes were related to follow-up reading attainment.

In this respect, and in agreement with previous studies on patients with diverse brain conditions including gliomas, we hypothesized that a compensatory rearrangement could occur at the white-matter level as well. We expected this compensation to involve the contralesional hemisphere, too, and be crucial to drive recovery from potential reading deficits resulting from tumor growth and/or surgery.

## Methods

### Study 1: Preoperative White-Matter Correlates of Reading Impairment

#### Participants

**Figure 1:**
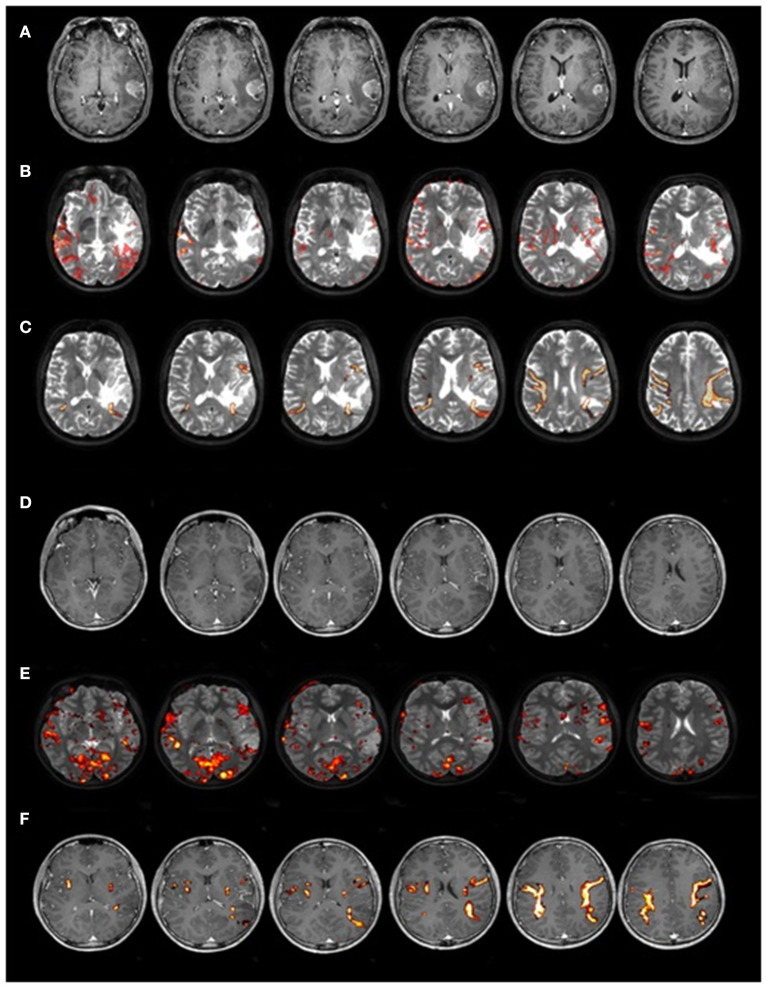
Two representative cases of patients with LGG and HGG. Magnetic resonance images of two patients with comparable lesion location from Study 1: one relatively circumscribed HGG (upper part of the image, **A–C**) and one LGG (lower part of the image, **D–F**). In **(A,D)** T1-weighted MRI image, in **(B,E)** fMRI map of a reading word task overlapped on T2-weighted MRI image, and in **(C,F)** DTI reconstruction of AF.

We tested a consecutive series of 40 patients [21 females; age (years): *M* = 41.70, *SD* = 11.58, range 16–68; mean handedness 91.82 ± 22.39%, following (24)^1^]. We included patients with both low-grade (LGG, *n* = 23/40) and high-grade (HGG, *n* = 17/40) left-hemisphere gliomas, which were almost anatomically circumscribed (see Figure 1 for an exemplificative case). We excluded patients with a family history of developmental language problems or learning disabilities. Subjects were all monolingual native speakers of Italian and had comparable levels (years) of education (*M* = 10.5, *SD* = 0.89, range 9–12, see Supplementary Table 1 for details).

Classification between LGGs and HGGs was first performed on a radiological basis (respectively, non-contrast-enhancing and contrast-enhancing MR images) and then confirmed by a histopathological diagnosis (LGGs included slowly growing grade I and II gliomas and HGGs included anaplastic gliomas and glioblastomas). In this analysis, we treated patients with LGG and HGG as a single group. Although the rationale for grouping LGG and HGG could be debatable, this study aimed to identify which fascicles—if already affected preoperatively and hence more vulnerable to damage by resection—can be responsible for an impaired reading performance postoperatively. To this end, the grade of the lesion affecting the fascicles of interest is irrelevant. Patients were selected serially; however, we ensured that patients with LGG and HGG were comparable in terms of lesion location or volume.

The preoperative assessment took place normally a few days before surgery, with neuropsychological testing (Supplementary Table 1) and neuroimaging session—including preoperative DTI—performed on the same day. In the immediate post-surgery period (1 week later), patients were re-tested to look for potential changes in their neuropsychological abilities and, in particular, in their reading skills (see below).

The patients' DTI data were also compared with those of a group of 20 healthy, monolingual Italian right-handed controls [nine females; age (years): *M* = 33.90, *SD* = 9.06, range 20–53; education (years): *M* = 16.07, *SD* = 1.75, range 13–18].

Participants signed an informed consent to participate in the study, in line with the Declaration of Helsinki. The study was approved by the local Ethics Committee.

#### Word and Pseudoword Reading Tasks

We used the reading aloud tasks from the battery for the analysis of language disorders (25). The battery included 92 stimuli for the *word reading task* and 45 stimuli for the *pseudoword reading task*. Following the authors' guidelines, the cutoff accuracy was 90/92 for the word reading task and 43/45 for the pseudoword reading task. Based on these cutoff scores, the patients' reading performance was classified as *impaired* or *spared*.

#### DTI Acquisition

A 3-T Philips Achieva whole-body scanner was used to acquire structural data using a SENSE-Head-8 channel head coil and a custom-built head restrainer to minimize head movements.

For both patients and healthy controls, diffusion tensor imaging (DTI) data were acquired using an axial diffusion-weighted, single-shot, echo-planar imaging sequence covering the whole brain (repetition time = 8,880 ms; echo time = 70 ms, bandwidth = 3,135 Hz/pixel; flip angle = 90°; matrix size = 128 × 128 voxels; field of view = 240 × 240 mm; slice thickness = 2.1 mm; contiguous axial slices = 57). Two *b*-values were used: 0 s/mm^2^ (seven no diffusion-weighted images) and 1,000 s/mm^2^ (64 non-coplanar, diffusion-weighted images). The 64 gradient directions were uniformly distributed on a sphere.

#### DTI Image Processing

We performed tract reconstruction by the DTISudio software (version 1.0.0.1) using the deterministic Fiber Assignment by Continuous Tracking (FACT) algorithm. Criteria for reconstruction were: Fractional anisotropy (FA) = 0.15, as a threshold for both starting and stopping tracking [see (26)], and a maximum turning angle = 70°. Given these permissive thresholds, tracked fascicles were then visually inspected in order to remove reconstructed fibers with an anomalous trajectory and, therefore, not anatomically plausible.

We reconstructed the following fascicles: Inferior Longitudinal Fasciculus (ILF), Inferior Fronto-Occipital Fasciculus (IFOF), Uncinate Fasciculus (UF), and Arcuate Fasciculus (AF). We also virtually segmented the AF, in order to inspect possibly different involvement of the diverse segments. In detail, we segmented the AF into anterior, posterior, and direct long segments according to Catani et al. (27). In addition, we followed the anatomical dissociation reported by Glasser and Rilling (28), in which the AF essentially corresponded to the direct long segment as intended by Catani et al. (27) and which was divided into a segment originating in the middle and inferior temporal gyrus (MTG) and another in the superior temporal gyrus (STG).

The main difference between the two tracing methods is that Catani et al. combined STG and MTG pathways into a single segment, arguing that the entire pathway conveys phonetic information to frontal lobe. Conversely, Glasser and Rilling showed that STG and MTG terminations connect cortices with different functions and suggested that they should be tracked as two separate pathways. In detail, the MTG segment overlapped with lexical-semantic activations and the STG segment with phonological activations, suggesting that they could correspond to the ventral and dorsal streams of Hickok and Poeppel's (29) model of speech processing, respectively.

Fascicles were reconstructed in both hemispheres by following the multi-ROI approach proposed by Catani et al. (27), Glasser and Rilling (28), and Catani and Thiebaut de Schotten (30). Hence, tract reconstruction was made by taking the same reference points for all the subjects, thus minimizing the effect of different brain sizes. We then drew the ROIs following the guidelines reported in these reference papers and used “AND” and “NOT” operators, respectively, to include fibers passing through both specified ROIs (e.g., anterior temporal and occipital ROIs for ILF, anterior temporal and external/extreme capsule ROIs for UF, occipital and external/extreme capsule ROIs for IFOF, inferior frontal and temporal ROIs for the AF Long segment, inferior frontal and inferior parietal ROIs for the AF Anterior segment, inferior parietal and temporal ROIs for the AF Posterior segment, posterior superior temporal and inferior frontal ROIs for the AF STG segment, and middle temporal and inferior frontal ROIs for the AF MTG segment) and to exclude fibers not belonging to the tract (e.g., frontal projections belonging to IFOF possibly reconstructed during ILF tracking).

Tracking was performed for clinical purposes by a medical physicist (M.M.) and then inspected by a team of neuroradiologists; these practitioners were both blind to information about the patients' reading performance. In detail, in the clinical routine, three neuroradiologists always inspect the reconstructed tracts before writing the MRI medical report. In case of disagreement, fibers were tracked again based on the anatomical atlas (31, 32) and starting from accurate detection by the same neuroradiologists of the above-mentioned reference points; finally, a further comparison was performed to check fascicle matching with the atlas. Bias associated with tracking was canceled out as a consistent tracking procedure was applied across all subjects by taking the same reference points for ROI identification (see above).

As a second step, reconstructed tracts were loaded to extract the DTI parameters of interest for each fascicle and fascicle segment (i.e., FA, and number, density, and length of streamlines, all computed by the DtiStudio software used for tracking) and statistical analyses were performed (E.C.).

DTI parameters were classified into two categories following the distinction made in previous papers [e.g., (33, 34)]. The first category included the number, density, and length of streamlines, which we defined as *macrostructural* parameters, as they are related to the macroscopic structural representation of fascicles themselves in the cerebral space; the second category included fractional anisotropy, trace, the three geometrical indices (linear, planar, and spherical), the three eigenvalues, mean diffusivity, and radial diffusivity, which are instead *microstructural* parameters, meaning scalar indices that reflect the within-voxel microscopic fiber structure [see (35, 36)]. Regarding both density and length of streamlines and scalar indices, the values we included in the analyses were the mean values of each tract.

### Study 2: Neuroplasticity Processes Occurring After Surgery

In order to inspect plasticity processes postoperatively, we longitudinally followed a subgroup of patients with LGG [*n* = 13, seven females; age (years): *M* = 34.62, *SD* = 10.24, range 16–52], which we retested at a follow-up assessment 4 months after surgery, when potential direct effects of surgery (e.g., brain swelling, edema) had resolved and potential plasticity processes could have taken place. Patients with HGG were excluded because of the postoperative clinical protocol including radio-chemotherapy, which might have an impact on both brain structures and cognitive status [e.g., (37)]. The follow-up assessment following preoperative assessment included both neuropsychological testing and DTI imaging acquisition (see Study 1).

### Statistical Analyses

#### Study 1: Preoperative White-Matter Correlates of Reading Impairment

Based on their 1-week post-surgery reading scores, we identified the two groups of patients with impaired and spared performance (see Results section for additional details) in either words or pseudowords.

We performed a multivariate analysis of variance (by using the SPSS 21.0 software, Inc., Chicago, IL) for the two groups of patients and healthy controls taking into account both DTI macrostructural parameters (fiber number, density, and length) and FA extracted from pre-surgery assessment. We included age as a covariate, together with gender. We also included tumor volume as a covariate, given that it significantly differed between patients with impaired and patients with spared reading performance (see below). Then, we carried out *post-hoc* comparisons (Bonferroni correction) in order to directly compare group pairs.

We computed tumor volume and extent of resection in order to inspect whether these variables significantly differed between patients with impaired vs. spared reading performance (and also between patients with LGG and those with HGG). Both lesion volume and extent of resection were routinely calculated by the neurosurgeon, who was blind to the purposes of the study. We included this information *a posteriori* for the purposes of the present study. Lesion volume was calculated by the MRIcro software based on the volumes of interest (VOIs) drawn on the patients' lesion. Extent of resection (EoR) was computed from tumor volumes on T2-MRI sequence for LGGs (38, 39), whereas post-gadolinium contrast T1 MRI sequences were used for HGGs [see (40, 41)]. All HGGs were enhancing tumors at preoperative MRI examination. This information was confirmed by histopathological analyses revealing that all lesions were grade III–IV tumors.

#### Study 2: Postoperative Reorganization in Patients With LGG

To inspect whether changes in DTI fascicle parameters had occurred at follow-up (i.e., 4 months after surgery), we compared follow-up to pre-surgery DTI parameters. As for many of these parameters normality assumption was violated, we performed the Wilcoxon non-parametric test.

Moreover, we investigated whether these significant changes correlated with follow-up reading performance by running an explorative non-parametric correlation analysis between DTI parameters and reading scores, both collected at the follow-up assessment.

Finally, for additional comparison, the same analyses were run on a white-matter bundle not involved in language, namely the cortico-spinal tract.

## Results

### Study 1: Preoperative White-Matter Correlates of Reading Impairment

#### Descriptive Results

Concerning preoperative reading performance, seven patients (six HGGs and one LGGs) were below the cutoff for word and/or pseudoword reading (see Supplementary Table 1). At immediate (1-week) post-surgery testing, 23 patients (14 females, 11 HGGs, and 12 LGGs) were below the cutoff and were therefore included in the group with *impaired* reading; conversely, 17 patients (seven females, six HGGs, and 11 LGGs) retained an above-cutoff reading performance and were then included in the group with *spared* reading.

**Figure 2:**
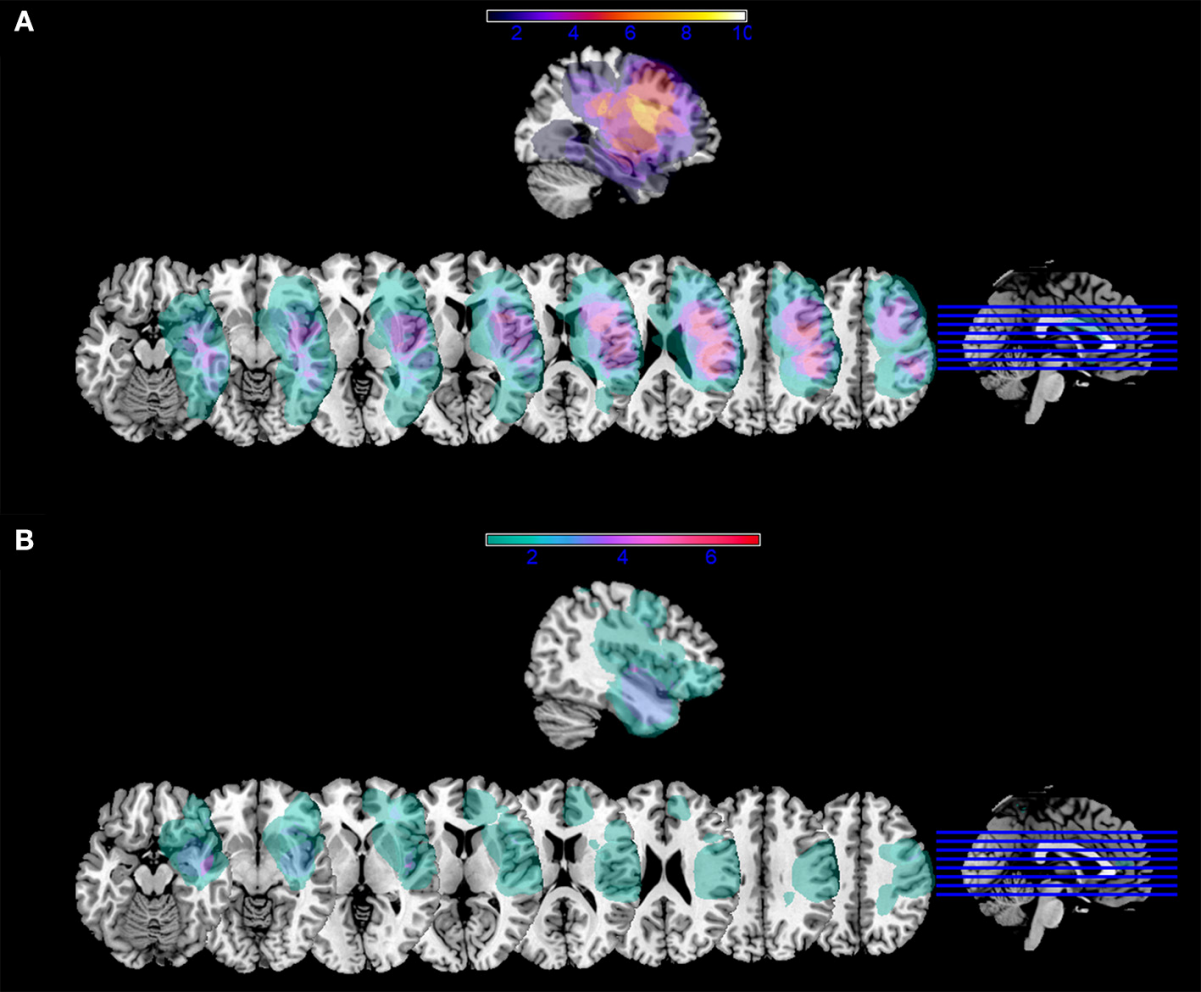
Lesion mask overlap. Overlap of lesion masks in **(A)** patients with impaired reading performance at one week; **(B)** patients with spared reading performance at one week. Color bars indicate the number of patients with lesion overlap in a given area. Images are shown in radiological convention.

We underline that, although it is known from the literature that reading words and reading pseudowords are supported by both shared and specific brain structures [e.g., (42, 43)], our data did not allow a differential diagnosis of specific deficits in either case. Further, more than half of the patients were impaired in both (*n* = 14/23) and there were only a few patients being impaired in either words (*n* = 3/23) or pseudowords (*n* = 6/23). In Figure 2, we reported lesion overlap separately for patients with impaired reading and patients with spared reading.

Importantly, we verified that immediate post-surgery reading performance did not significantly differ between patients with LGG and HGG (words: *M*_LGG_−*M*_HGG_ = 3.20, *t* = 1.13, *p* = 0.27; pseudowords: *M*_LGG−_ *M*_HGG_ = 0.55, *t* = 0.21, *p* = 0.84). This confirmed that, for localization purposes, they could be treated as a single group (see Supplementary Tables 1, 2 for details). Further, the two groups did not significantly differ in mean tumor volume (*t* = −0.74, *p* = 0.47), which was 53.25 mm^3^ (*SD* = 47.51 mm^3^) for LGGs and 67.60 mm^3^ (*SD* = 75.76 mm^3^) for HGGs. Mean EoR was 88.67% (*SD* = 18.46%) for LGGs and 92.50% (SD = 11.83%) for HGGs and was not significantly different between the two groups (*t* = −0.73, *p* = 0.47).

Nevertheless, as anticipated, tumor volume (but not EoR) significantly differed between patients with impaired vs. spared reading performance (*t* = 2.05, *p* = 0.05): 73.85 mm^3^ (*SD* = 69.40 mm^3^) and 38.88 mm^3^ (*SD* = 37.13 mm^3^), respectively. However, in neither group was tumor volume significantly related with the 1-week reading performance on words or pseudowords. As mentioned earlier on, we therefore included tumor volume as a covariate, together with age.

In point of fact, even though age did not significantly differ between patients with impaired reading and those with spared postoperative reading (*t* = 1.66, *p* = 0.11), it was significantly different between patients and healthy controls (*t* = 2.63, *p* = 0.01). In order to exclude potential bias in the results because of significantly different age, we inspected the FA value distribution in relation to age separately for patients and healthy controls (see Supplementary Figure 1). It is possible to observe that there is not a clear trajectory depending on the subject's age; this was particularly true for patients for whom lesion may have a more relevant impact than age. In addition, the majority of both patients and controls were in their 30–40s, an age range during which no marked changes are reported in FA values: around this age, FA reaches its peak and then slowly decreases [e.g., (44)].

#### Comparison Between Patients With Impaired Reading and Patients With Spared Reading (Δ*M* = *M*_spared_ – *M*_impaired_)

A first exploratory analysis showed that the most salient and informative parameters were the three macrostructural parameters (i.e., fiber number, density, and length) and fractional anisotropy (FA), among the microstructural ones. We therefore retained these parameters for the analyses we performed.

**Figure 3:**
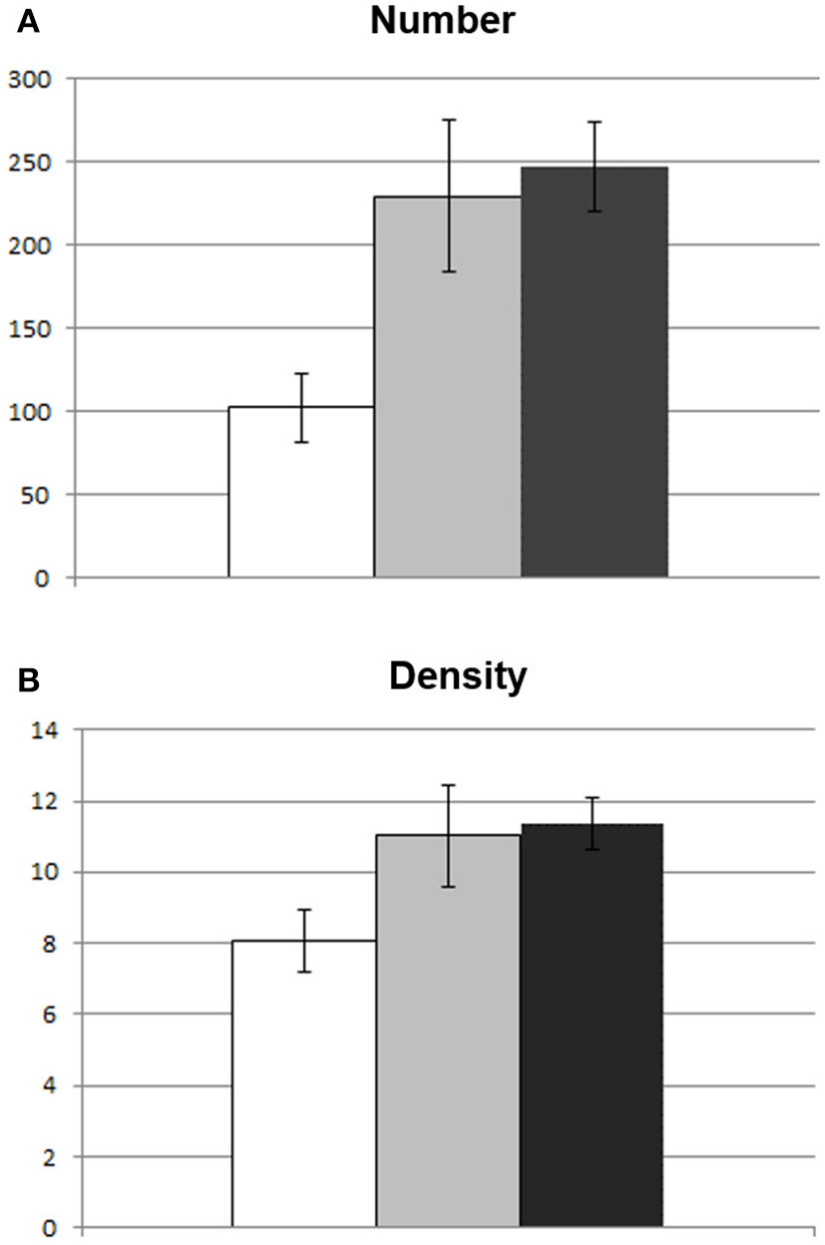
Between-group differences in macrostuctural DTI parameters of the AF MTG segment. Graphic representation of difference in mean DTI parameters between patients with impaired reading (white) and those with spared reading (light gray) at one week. Healthy control subject mean values are also represented (dark gray). In **(A)** difference in number of streamlines; **(B)** difference in density (number of streamlines/voxel). Bars represent standard error of the mean.

Concerning preoperative DTI macrostructural parameters, the group with impaired reading had significantly lower values of number and density of streamlines of the AF MTG segment in the left hemisphere (see Figure 3). In the right hemisphere, none of the parameters significantly differed between the two groups (see Table 1).

**Table 1:** Between-group differences in macrostructural DTI parameters.

	**Δ*M = M*_**spared**_**−***M*_**impaired**_**	**Δ*M* = *M*_**healthy**_**−***M*_**impaired**_**	**Δ*M = M*_**healthy**_**−***M*_**spared**_**
**LH**			
UF	*n.s*.	Length: Δ*M* = 13.05, *p* = 0.023	*n.s*.
AF Long	*n.s*.	Length: *ΔM* = 12.63, *p* = 0.041	*n.s*.
MTG	Number: *ΔM* = 154.25, *p* = 0.026	Number: *ΔM* = 175.06, *p* = 0.029	*n.s*.
		Density: Δ*M* = 4.86, *p* = 0.045	

Comparison of macrostructural DTI parameters (i.e., number, density, and length of streamlines) between patients with impaired and spared performance (ΔM = M_spared_−M_impaired_), between patients with impaired performance and healthy controls (ΔM = M_healthy_−M_impaired_), and between patients with spared performance and healthy controls (ΔM = M_healthy_−M_spared_).

LH, left hemisphere; RH, right hemisphere; AF, arcuate fasciculus; MTG, part of the arcuate fasciculus originating in the middle temporal gyrus; UF, uncinate fasciculus.

*Length is expressed in mm and density in number of streamlines per voxel*.

The preoperative DTI microstructural parameter (FA) were significantly lower in patients with impaired reading for almost all fascicles of the left hemisphere (i.e., ILF, IFOF, UF, AF Anterior segment, and AF MTG; see Figure 3) and for none of the fascicles of the right hemisphere (see Table 2).

**Table 2:** Between-group differences in microstructural DTI parameters.

	**Δ*M = M*_**spared**_**−***M*_**impaired**_**	**Δ*M* = *M*_**healthy**_**−***M*_**impaired**_**	**Δ*M = M*_**healthy**_**−***M*_**spared**_**
**LH**			
ILF	Δ*M* = 0.029, *p* = 0.020	Δ*M* = 0.045, *p* = 0.001	*n.s*.
IFOF	Δ*M* = 0.039, *p* = 0.017	Δ*M* = 0.044, *p* = 0.012	*n.s*.
UF	Δ*M* = 0.047, *p* = 0.006	Δ*M* = 0.055, *p* = 0.002	*n.s*.
AF Anterior	Δ*M* = 0.026, *p* = 0.012	*n.s*.	*n.s*.
AF Posterior	*n.s*.	Δ*M* = 0.044, *p* = 0.006	*n.s*.
AF Long	*n.s*.	Δ*M* = 0.057, *p* = 0.011	*n.s*.
MTG	Δ*M* = 0.044, *p* = 0.008	Δ*M* = 0.061, *p* = 0.001	*n.s*.
STG	*n.s*.	*n.s*.	*n.s*.
**RH**			
ILF	*n.s*.	*n.s*.	*n.s*.
IFOF	*n.s*.	*n.s*.	*n.s*.
UF	*n.s*.	Δ*M* = 0.024, *p* = 0.031	*n.s*.
AF Anterior	*n.s*.	Δ*M* = 0.035, *p* = 0.018	*n.s*.
AF Posterior	*n.s*.	Δ*M* = 0.034, *p* = 0.005	*n.s*.
AF Long	*n.s*.	Δ*M* = 0.032, *p* = 0.018	*n.s*.
STG	*n.s*.	*n.s*.	*n.s*.
MTG	*n.s*.	Δ*M* = 0.035, *p* = 0.019	*n.s*.

Comparison of microstructural DTI parameter (i.e., Fractional Anisotropy, FA) between patients with impaired and spared performance (ΔM = M_spared_−M_impaired_), between patients with impaired performance and healthy controls (ΔM = M_healthy_−M_impaired_), and between patients with spared performance and healthy controls (ΔM = M_healthy_−M_spared_).

*LH, left hemisphere; RH, right hemisphere; AF, arcuate fasciculus; IFOF, inferior fronto-occipital fasciculus; ILF, inferior longitudinal fasciculus; MTG, part of the arcuate fasciculus originating in middle temporal gyrus; STG, part of the arcuate fasciculus originating in superior temporal gyrus; UF, uncinate fasciculus*.

#### Comparison Between Patients With Impaired Reading and Healthy Controls (Δ*M* = *M*_healthy_ – *M*_impaired_)

In the left hemisphere, fascicles differing in terms of DTI macrostructural parameters between patients with impaired reading and healthy controls were (see Table 1): UF (mean length of streamlines), AF Long segment (mean length of streamlines), and AF MTG segment (number and density of streamlines); in the right hemisphere, no significant differences were observed.

The DTI microstructural parameter (FA) was significantly lower for patients with impaired reading vs. healthy controls in several fascicles of both the left hemisphere (i.e., ILF, IFOF, UF, AF Posterior segment, AF Long segment, and AF MTG segment) and the right hemisphere (i.e., UF, AF Anterior, AF Posterior segment, AF Long segment, and AF MTG segment) (see Table 2).

Differences (non-significant) between patients with spared reading and healthy controls are also reported in Tables 1, 2.

### Study 2: Postoperative Reorganization in Patients With LGG

#### Reading Performance

Neuropsychological data showed that patients' reading performance improved vs. the 1-week post-surgery assessment (see Supplementary Tables 1, 2). Hence, we wanted to inspect whether there were significant changes in DTI fascicle parameters and whether these related with improved reading.

#### Changes in DTI Parameters Between Pre-surgery and Follow-Up

**Figure 4:**
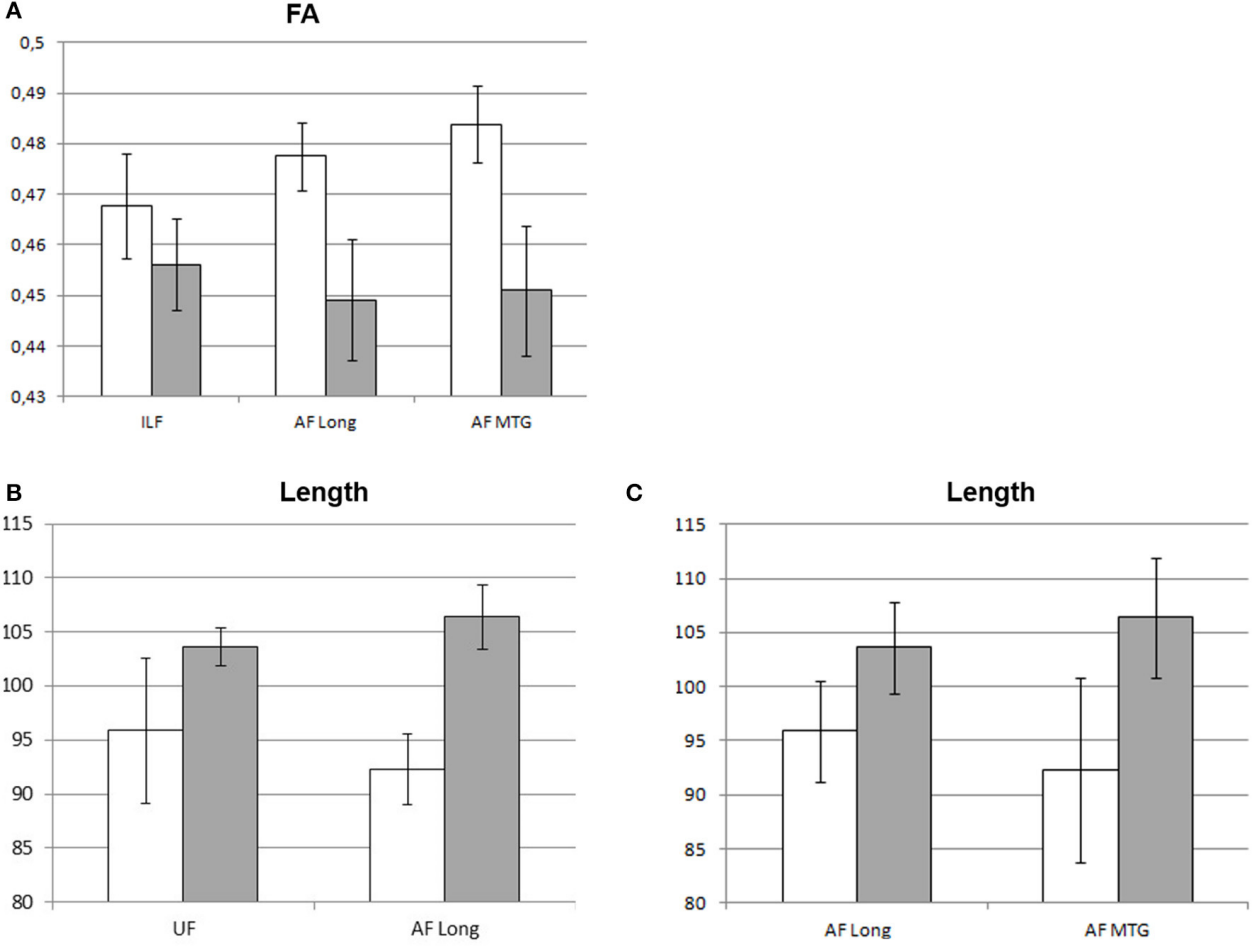
Significant changes in mean DTI parameters between pre-surgery and follow-up. Graphic representation of the DTI parameters showing significant changes at follow-up (gray) vs. pre-surgery (white): **(A)** FA values (left hemisphere); **(B)** left-hemisphere length (mm) of streamlines; **(C)** right-hemisphere length (mm) of streamlines. Bars represent standard error of the mean.

At the 4-month follow-up, we found a decrease in FA values of ILF, AF Long segment, and AF MTG segment in the left hemisphere and no significant decreases in the right hemisphere. As regards findings that could instead be interpreted in a compensatory plasticity perspective, we found a significant increase in length of streamlines of the UF and AF Long segment in the left hemisphere and of AF Long and AF MTG segments in the right hemisphere (see Table 3 and Figure 4).

**Table 3:** Significant changes in DTI parameters between follow-up and pre-surgery.

	**Parameter**	**Difference**	***z*-value**	***p***
**LH**				
ILF	FA	−0.01	−1.96^a^	0.05
UF	Length	8.11	−2.07^b^	0.04
AF Long	FA	−0.03	−2.85^a^	<0.01
	Length	7.02	−2.22^b^	0.03
AF MTG	FA	−0.03	−2.93^a^	<0.01
**RH**				
AF Long	Length	7.38	−2.31^b^	0.02
AF MTG	Length	14.19	−2.05^b^	0.04

LH, left hemisphere; RH, right hemisphere; AF, arcuate fasciculus; ILF, inferior longitudinal fasciculus.

a z-value based on the positive ranks;

b *z-value based on the negative ranks*.

The non-parametric correlation analysis between the parameters showing a significant change at follow-up (i.e., FA and length of streamlines of the mentioned fascicles) and follow-up reading scores did not show any results reaching statistical significance.

Finally, the additional comparison involving the cortico-spinal tract did not show any significant changes in DTI parameters between pre-surgery and follow-up nor significant correlations with reading performance (see Supplementary Tables 3, 4).

## Discussion

In this study, we investigated the link between white matter tracts—arcuate fasciculus in particular—and reading abilities in a population of patients undergoing surgery for a glioma. To this end, we explored the white-matter role in reading attainment by investigating which fascicles were related with the reading impairment some patients developed in the acute postoperative period (Study 1). Second, we addressed the extent of plasticity processes 4 months after surgery in patients with LGG specifically; we hence inspected whether potential changes in the same white-matter fascicles could be related to improved follow-up reading skills (Study 2).

### Clinical Observations

In Study 1, we included patients with both LGGs and HGGs (Study 1). In Study 2, the analysis of follow-up data was focused on patients with LGG. The rationale for including patients with both LGGs and HGGs in Study 1 was based on the purpose to address subcortical correlates of reading in patients with glioma. In both groups, the white matter could be structurally affected as early as preoperatively, implying that resection had greater potential to damage these fascicles, causing a reading impairment accordingly. Conversely, we excluded patients with HGG from Study 2 because they underwent postoperative radio-chemotherapy treatments, which might affect both brain structures and cognitive performance and hence, bias the results. Patients with LGG, on the contrary, are suitable to explore postoperative brain reorganization. The aim of surgery is to remove the tumor mass and infiltrated parenchyma as well. It is indeed known that, the larger the resection, the longer the survival; this is very relevant, considering that patients with LGG are usually young and can survive several years after surgery. However, cognitive deficits may develop after surgery. For this reason, we explored if plasticity processes took place in the postoperative period and if they were eventually related to follow-up reading performance.

### Study 1: Preoperative White-Matter Correlates of Reading Impairment

To comment our results, we based on the assumption that the comparison between patients with impaired reading vs. spared postoperative reading would be most specific in indicating the neural correlates of reading. Conversely, the comparison between patients and healthy controls could only show the effect of tumor growth on white-matter fascicle parameters and was therefore independent of the reading performance status.

The specific comparison between patients with impaired postoperative reading and patients with preserved reading showed that a reading deficit was more likely to occur in the immediate postoperative phase if white-matter fascicles were affected as early as preoperatively. Figure 2 shows that lesion overlap in patients with impaired performance occurred in perisylvian areas and, in particular, in the inferior frontal gyrus. On the other hand, maximum lesion overlap for patients with spared reading occurred in the anterior and inferior temporal areas. Consistent with lesion overlap findings, a selective comparison of macrostructural DTI parameters between the two patient groups shows that the only fascicle being specifically more affected in the group with impaired reading was the left AF MTG segment above all, in terms of number and density of streamlines.

This segment is the most notable component of the AF Long segment, which directly connects temporal and frontal areas and is part of the so-called dorsal phonological route (45). In particular, the AF MTG segment directly connects the middle and inferior temporal cortex with frontal areas and seems crucial for conveying lexico-semantic information [see (28)]. On the other hand, we did not find relevant differences in the other AF Long component, meaning the AF STG segment, which links the posterior part of the superior temporal gyrus (Wernicke's area) with frontal regions and is more likely involved in phonological processing [see (28)]. However, we cannot exclude a potential role of this segment as well. In fact, reconstruction of this tract is challenging even in healthy subjects [see (28)] and might be further hindered by the lesion. In point of fact, we could not find it in 12 patients (nine of whom with impaired reading); this suggests that the AF STG segment as well may play a potential role in reading, but missed reconstruction in several patients could have prevented achievement of statistically relevant results.

To the best of our knowledge, previous studies did not explore the role of these segments in reading. However, Tomasino et al. (46) provided evidence of an association between preserved AF MTG segment and unaffected reading in a patient operated on for an LGG in the left superior and middle temporal lobe. Instead, a larger number of reports linked the AF Long segment with reading impairment, yet in other clinical populations: this fascicle was affected in individuals with lower reading skills [e.g., (47)] and developmental dyslexia [e.g., (8)]. Hence, although the AF has been not typically associated with reading [see (27)], our results support a crucial role for the direct AF segments connecting temporal and frontal cortex. In detail, our study suggests that fiber integrity of the left AF MTG segment—if already endangered by tumor growth—may be further affected by surgery and therefore trigger postoperative reading deficits.

FA values were significantly lower in almost all the left hemisphere fascicles in patients with impaired vs. spared reading. This finding indicates the higher susceptibility of this parameter and, hence, the fact that microstructural changes are likely to take place all over the entire affected hemisphere.

Differences in FA values indicate differences in anisotropy, meaning in coherent diffusion of water molecules, which, in normal conditions, is constrained along the axon direction (48). If pathological conditions are present, including the specific case of gliomas [e.g., (49, 50)], lower FA is frequently detected and indicates some kind of damage (e.g., demyelinization, alteration in fiber orientation, decreased coherence of fiber alignment). Caution is however needed when interpreting these findings in terms of white-matter disruption [see (51)]. Further, in a few studies, FA was selectively affected in patients developing deficits because of glioma vs. patients without deficits [e.g., (26)].

A specific comparison between the two patient groups did not reveal significant differences in the right hemisphere. A few differences only emerged in the FA values of some contralesional fascicles when patients with impaired—but not spared—reading performance were compared with healthy controls. Although healthy homologs are typically taken as reference to investigate the degree of impairment of ipsilesional fascicles, this finding indicates that the effects of tumor growth can extend to the healthy hemisphere as well [e.g., (52)] and be more marked in patients who then develop a postoperative reading impairment.

### Study 2: Postoperative Reorganization in Patients With LGG

At follow-up, reading was in the normal range for all patients with LGG we longitudinally monitored except one whose performance however improved vs. the 1-week assessment. Concerning white-matter fascicles, comparison of pre-surgery and follow-up DTI parameters showed a few significant changes. In detail, we found a significant decrease at follow-up in the FA values of ILF, AF Long segment, and AF MTG segment in the affected left hemisphere. Nevertheless, we also found a significant increase in length of streamlines of UF and AF Long segment in the left hemisphere and of AF Long and AF MTG segments in the right hemisphere.

A decrease in the FA values was partly unexpected, given the observed follow-up improvement in reading. This decrease was evocative of a lasting alteration in diffusion processes within the fibers. We do not believe that this decrease is attributable to edema, as potential postoperative edema had resolved 4 months after surgery [e.g., (6)] and edema potentially associated with residual tumor was not present as LGGs—given their slow-growing nature—do not cause the same edema mass effects as HGGs [e.g., (53–55)]. Decrease in FA was not accompanied by a decrease in macrostructural DTI parameters, suggesting that surgery had not disrupted these fibers. Therefore, it is conceivable that other surgery-related effects, like mechanical traction, could be still affecting some brain structures. Alternatively—or in addition—tumor resection allowed fiber reconstruction in regions where tracking had previously stopped. In some patients, however, potential residual tumor in these regions could still infiltrate white-matter tissue and, although not preventing tracking, it could contribute to lowering the mean FA value of the entire fascicle. If this were the case, the FA decrease is not in contradiction with improved reading skills and is congruent with the observed increase in streamline length of a few fascicles in both hemispheres.

Accordingly, increased length in the left hemisphere could be attributed to tumor removal, which allowed tracking of fibers that previously had been most likely masked by the tumor. Increase in contralesional hemisphere could not be interpreted in the same way but, possibly, in terms of plasticity. It has been observed that brain insults may determine recruitment of contralesional homologs via reduction of the interhemispheric inhibition exerted by the corpus callosum [e.g., (56, 57)]. Although plasticity mechanisms potentially occurring at white-matter level have not been clarified yet, it is possible that translocation of functional activations to contralesional cortical areas entails an increased communication through the fascicles connecting them and possibly unmasking of previously silent pathways. The increased fascicle length we observed did not mean that fibers were actually longer at follow-up, but this finding can reflect biochemical processes determining structural changes which, consequently, improved fascicle reconstruction.

Changes in streamline length notably involved AF Long and AF MTG segments. This finding confirms the leading role of direct AF segments observed in Study 1. Unfortunately, the small sample size did not enable us to carry out specific analyses to demonstrate the causal link between DTI changes and improved reading, and correlation analysis with reading scores did not provide any significant results.

Nevertheless, data from stroke experience [e.g., (58)] showed that the right AF Long segment significantly predicted recovery from aphasia after a left-hemisphere stroke. To the best of our knowledge, studies involving neurosurgical patients with glioma did not inspect the relation between postoperative reading performance and right AF segment remodeling. Preoperatively, Jehna et al. (59) observed that patients with either symmetric or right-lateralized AF segments displayed fewer language deficits than patients with left-sided lateralization. Although these findings could reflect pre-clinical lateralization and are not informative of the effect of surgery on right AF rearrangement, they however corroborate the importance of this contralesional fascicle in preserving language functions, including reading. Further investigation is necessary to inspect the possible role of the right AF Long segment in determining preserved or recovered reading following glioma resection, too.

## Limitations of the Study and Conclusions

Overall, findings from this study show the important role played by AF segments directly connecting the temporal lobe with frontal lobe in preserving reading skills in neurosurgical patients. In fact, their pre-surgical status could be useful to predict the possible development of postoperative reading deficits and subsequent improved connectivity through the same fascicles, including contralesional homologs, could drive recovery of reading deficits.

This study presents a few limitations, specifically, concerning the DTI methodology. Different tracking approaches exist, although there is no consensus yet on the most suitable protocol to adopt. This was a retrospective study and the applied DTI acquisition protocol did not enable to carry out more sophisticated tracking procedures. We used a deterministic algorithm for tracking, which has the major limit of preventing reconstruction of fiber branches. However, contrary to probabilistic algorithms, it uses an FA threshold, which prevents tracking in regions with very low anisotropy and reduces the probability of false positives [see (60, 61)]. A second important limitation concerned numerosity of patients in Study 2, which limited more thorough analysis and investigation of the actual role of white-matter changes in recovered reading performance.

Findings from the present study are nonetheless important from a neurosurgical perspective. In addition to evidence showing the need to preserve posterior ILF in order to prevent the onset of permanent reading deficits [e.g., (4)], current results also suggest the clinical importance of sparing AF direct segments. However, a potential compensatory reorganization at this level, including involvement of the contralesional homolog, as well, has to be better elucidated. These findings therefore need to be replicated by different tracking approaches and wider patient samples. Future studies should compare patients always retaining a good reading performance vs. patients with impaired reading immediately postoperatively who then recovered vs. patients who did not recover at all, in order to inspect potential differences in their white-matter status. A wider sample could also allow exploring the potentially different role of AF segments in reading words or pseudowords.

## Data Availability Statement

The raw data supporting the conclusions of this article will be made available by the authors, without undue reservation.

## Ethics Statement

The studies involving human participants were reviewed and approved by Comitato Etico Regionale Ceur FVG. Written informed consent to participate in this study was provided by the participants'Elegal guardian/next of kin.

## Author Contributions

EC: data analysis and curation, writing original draft, and writing review and editing. MM: tracking. TI: validation and writing review and editing. MS: supervision, validation, and writing review and editing. BT: supervision, data curation, validation, and writing review and editing. All authors contributed to the article and approved the submitted version.

## Conflict of Interest

The authors declare that the research was conducted in the absence of any commercial or financial relationships that could be construed as a potential conflict of interest.

## Footnotes

^1^Handedness as measured by the Edinburgh Inventory has possible values within the range of −100 (perfect left-handedness) to 100 (perfect right-handedness) and were expressed in percentage.
