# Supplementary material for: Relation Between Reading Performance and White-Matter Alteration and Reorganization in Neurosurgical Patients
Source: Front Neurol. 2020 Dec 14;11:563259. doi: 10.3389/fneur.2020.563259 (PMC7793733; doi:10.3389/fneur.2020.563259)
Supplement: Supplementary file 1 [file Data_Sheet_1.pdf]

Supplementary Table S1. Patients' demographic data and neuropsychological performance

Pt	Sex	Age	Edu	Hand	Localization	Diagnosis*	Vol (cc)	EoR (%)	Raven (/36)	OA (/20)	IMA (/72)	Token (/36)	BADA (/30)	Pre W (/92)	Pre PW (/45)	One-week W (/92)	One-week PW (/45)	F-up W (/92)	F-up PW (/45)
1	f	33	17	100	T Pm IFG	Oligodendroglioma, IDH mutant and 1p/19q codeleted	170.81	45	35	20	70	32	30	92	45	**83**	**31**	n/a	n/a
2	m	35	13	100	T ins	Diffuse astrocytoma, IDH mutant	43.48	100	34	20	72	34	29	92	45	**86**	45	92	45
3	m	24	13	100	TP	Glioblastoma	44.62	75	34	19	61	**25**	28	**86**	**38**	**72**	**32**	n/a	n/a
4	f	33	18	100	T Pm ins IFG	Anaplastic astrocytoma	208.61	75	36	20	72	36	29	92	45	**86**	45	n/a	n/a
5	f	52	13	100	Pm IFG	Oligodendroglioma, IDH mutant and 1p/19q codeleted	109.11	100	30	20	71	32	29	92	44	**57**	**13**	92	45
6	f	53	8	100	TP	Glioblastoma	45.5	70	30	20	72	34	30	92	43	**60**	**33**	n/a	n/a
7	f	38	18	100	T ins F	Anaplastic astrocytoma, IDH mutant	122.84	85	34	20	70	34	29	91	45	**84**	**40**	n/a	n/a
8	m	15	11	100	T	Dysembryoplastic neuroepithelial tumour	20.76	65	35	20	70	36	**26**	92	44	**82**	**40**	**89**	**41**
9	f	50	13	100	T Pm	Glioblastoma	63.94	100	36	20	53	30	29	92	**38**	**85**	**28**	n/a	n/a
10	m	39	13	100	T	Diffuse astrocytoma, IDH mutant	49.16	100	34	20	72	36	30	92	44	**82**	**37**	n/a	n/a
11	m	45	13	100	TP	Oligoastrocytoma	41.89	100	27	20	72	36	30	92	44	92	**41**	n/a	n/a
12	f	33	8	100	TP	Glioblastoma	20.3	100	26	20	72	34	27	**85**	**40**	**67**	**38**	n/a	n/a
13	f	58	13	100	TO	Glioblastoma	35.57	100	32	20	72	34	29	**72**	**31**	**73**	**36**	n/a	n/a
14	m	53	8	100	P	Glioblastoma	57.68	100	28	20	**52**	29	30	90	**38**	**86**	**36**	n/a	n/a

15	f	50	13	100	F ins	Anaplastic astrocytoma	282.04	100	29	20	63	28	**26**	90	44	92	**42**	n/a	n/a
16	m	57	11	100	P	Glioblastoma	39.93	100	29	20	67	n/a	30	92	**40**	90	**26**	n/a	n/a
17	m	44	18	100	Pm	Diffuse astrocytoma	55.83	65	36	20	71	36	30	92	45	92	**39**	n/a	n/a
18	f	61	18	100	T	Diffuse astrocytoma, IDH mutant	16.94	92	35	20	72	34	30	92	45	**85**	**11**	n/a	n/a
19	f	31	17	90	T	Oligodendroglioma, IDH mutant and 1p/19q codeleted	31.4	91	33	20	72	36	**18**	**88**	45	90	**41**	92	45
20	m	42	13	95	T	Oligodendroglioma, IDH mutant and 1p/19q codeleted	16.51	87	36	20	72	36	30	92	45	**83**	44	n/a	n/a
21	f	67	8	100	F Pm IFG	Glioblastoma	59.21	100	29	20	68	**27**	**26**	92	44	92	**39**	n/a	n/a
22	f	34	13	96,66	F ins	Diffuse astrocytoma, IDH mutant	152.25	100	30	20	72	36	29	92	43	**86**	**35**	n/a	n/a
23	f	47	8	100	F post	Diffuse astrocytoma	22.9	100	36	20	72	**26**	29	91	45	**79**	**40**	92	**43**
24	f	62	13	55	Pm	Glioblastoma	11.66	100	34	20	72	35	30	92	45	92	45	n/a	n/a
25	m	48	11	100	F	Pilocytic astrocytoma	50.49	100	28	20	69	35	30	92	45	92	45	n/a	n/a
26	f	32	17	50	T ins	Diffuse astrocytoma, IDH mutant	30.8	100	36	20	72	36	29	91	45	92	45	92	45
27	f	28	13	100	IFG	Diffuse astrocytoma, IDH wildtype	1.48	100	34	20	70	36	30	92	45	91	45	92	45
28	f	48	18	100	T	Oligodendroglioma, IDH mutant and 1p/19q codeleted	12.25	100	33	20	72	36	30	92	45	92	45	n/a	n/a
29	m	39	18	100	F	Glioblastoma	15.8	100	35	20	72	36	30	92	45	92	45	n/a	n/a
30	m	40	13	100	IFG	Oligoastrocytoma	7.12	100	32	20	72	36	30	92	45	92	45	92	45
31	f	53	13	100	P	Glioblastoma	43.3	100	31	20	72	36	30	92	45	92	45	n/a	n/a
32	f	19	13	100	T ins	Diffuse astrocytoma, IDH	16.64	100	28	20	72	36	30	92	44	91	45	92	45

						wildtype												
33	f	43	13	10	PFC	Oligodendroglioma, IDH mutant and 1p/19q codeleted	35.5	100	35	20	72	35	30	92	45	92	45	92	45
34	m	50	13	100	F	Glioblastoma	21.3	75	29	20	72	36	30	92	45	92	45	n/a	n/a
35	m	40	13	100	Pm F	Glioblastoma	9.2	100	30	20	72	35	30	92	45	90	45	n/a	n/a
36	m	38	13	83.33	F IFG	Diffuse astrocytoma, IDH mutant	59.56	98	30	20	70	35	30	92	44	92	45	92	45
37	m	38	18	100	F	Ganglioglioma	23.3	90	33	20	72	35	30	92	45	92	45	n/a	n/a
38	m	49	8	100	T ins	Oligodendroglioma, IDH mutant and 1p/19q codeleted	78.68	50	31	18	72	36	28	92	45	91	44	n/a	n/a
39	m	33	8	100	FT ins	Oligoastrocytoma	145.47	50	26	20	71	32	27	92	45	92	44	91	44
40	m	32	18	50	Ins	Diffuse astrocytoma, IDH mutant	85.62	95	34	20	72	36	30	92	45	92	45	91	44

Note. * Diagnosis reports the most recent 2016 WHO classification (which includes molecular markers) for those patients for whom it was available. Edu: years of education; EoR: extent of resection; F: frontal; FT: fronto-temporal; f-up: follow-up; Hand: handedness; IFG: inferior frontal gyrus; IMA: ideomotor apraxia; ins: insula; OA: oral apraxia; P: parietal; PFC: prefrontal; Pm: premotor; Pt: patient's ID; PW: pseudoword reading; STM: short-term memory; T: temporal; vol: volume; TO: temporo-occipital; TP: temporo-parietal; W: word reading. Numbers in bold font indicate when the performance was below the cutoff.

Supplementary Table S2. Number of patients with impaired reading of words, pseudowords, or both in the three different assessment times.

	Words	Pseudowords	Both	Total
**Pre-surgery**	2/40	3/40	2/40	7/40
**One week**	3/40	6/40	14/40	23/40
**Follow-up**	0/13	0/13	1/13	1/13

Supplementary Table S3. Difference in the DTI parameters of the cortico-spinal tract between follow-up and pre-surgery.

	Parameter	Difference	*z*-value	*p*
**LH**	Number	10.07	-1.18[a]	.24
	Density	5.81	-0.38[a]	.70
	Length	-3.61	-1.78[a]	.75
	FA	.01	-1.57	.11
**RH**	Number	1.77	-.71[a]	.48
	Density	-0.35	-0.94[b]	.35
	Length	-5.07	-1.01[a]	.71
	FA	< .01	-0.04[b]	.97

Note. LH = left hemisphere; RH = right hemisphere. Density is expressed in number of streamlines/voxels and FA in mm. [a] *z*-value based on the negative ranks; [b] *z*-value based on the positive ranks.

Supplementary Table S4. Bivariate non-parametric (Spearman) correlations between DTI parameters of the cortico-spinal tract and postoperative scores in word and pseudoword reading.

	Word reading	Pseudoword reading
**LH**		
Number	.04 (*p* =.89)	.17 (*p* =.59)
Density	-.02 (*p* = .92)	.25 (*p* = .44)
Length	-.13 (*p* = .68)	.13 (*p* = .69)
FA	.26 (*p* = .41)	.07 (*p* = .82)
**RH**		
Number	.38 (*p* =.21)	.48 (*p* =.10)
Density	.37 (*p* = .22)	.41 (*p* = .16)
Length	-.06 (*p* = .85)	-.09 (*p* = .77)
FA	.09 (*p* = .79)	.13 (*p* = .69)

Note. LH = left hemisphere; RH = right hemisphere. Density is expressed in number of streamlines/voxels and FA in mm.

Supplementary Figure 1. FA value distribution in relation to age.

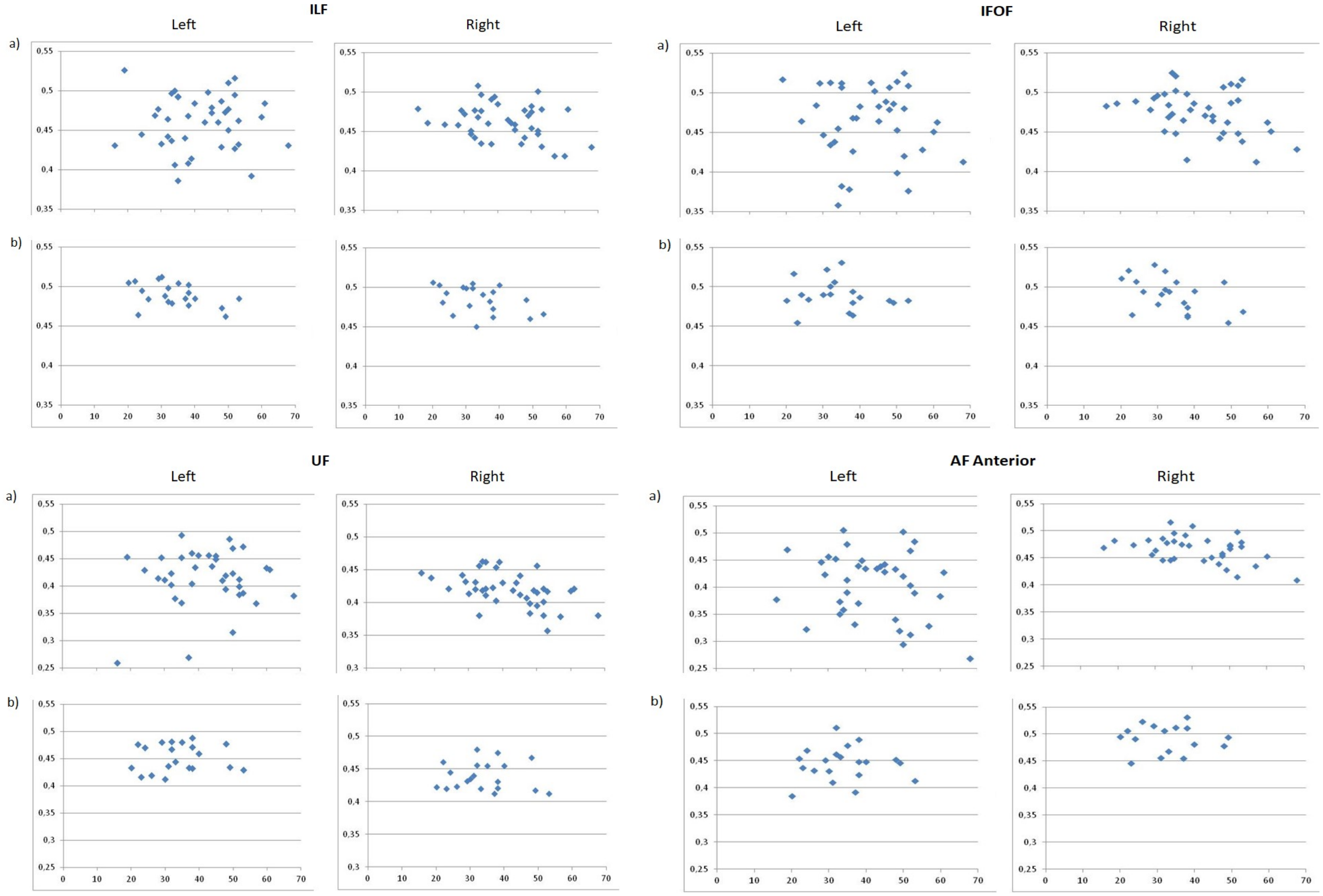

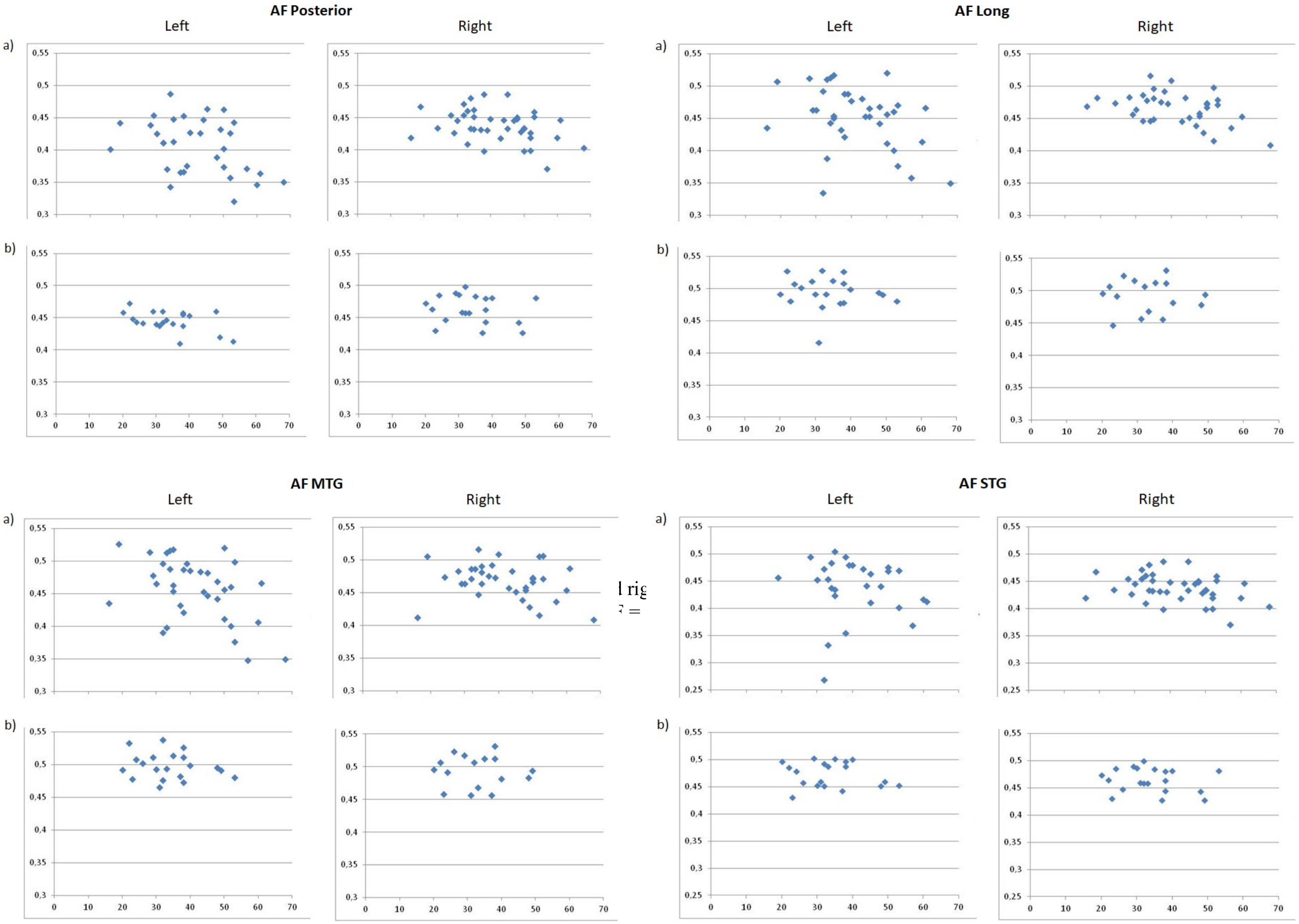
AF Posterior
Left
Right
a)
b)
AF Long
Left
Right
a)
b)
AF MTG
Left
Right
a)
b)
AF STG
Left
Right
a)
b)
